# Deep-Sea Sediments from the Southern Gulf of Mexico Harbor a Wide Diversity of PKS I Genes

**DOI:** 10.3390/antibiotics11070887

**Published:** 2022-07-04

**Authors:** Maikel Fernández-López, Ayixon Sánchez-Reyes, Clara Barcelos, Karla Sidón-Ceseña, Ricardo B. Leite, Asunción Lago-Lestón

**Affiliations:** 1 Centro de Investigación en Dinámica Celular, Instituto de Investigación en Ciencias Básicas y Aplicadas, Universidad Autónoma del Estado de Morelos, Av. Universidad 1001, Col. Chamilpa, Cuernavaca 62209, Mexico; fmaikel44@gmail.com; 2 CONACYT-Instituto de Biotecnología, Universidad Nacional Autónoma de México (UNAM), Av. Universidad 2001, Col. Chamilpa, Cuernavaca 62210, Mexico; ayixon.sanchez@mail.ibt.unam.mx; 3 Posgrado de Ciencias de la Vida, Centro de Investigación Científica y de Educación Superior de Ensenada, Carretera Ensenada-Tijuana No. 3918, Zona Playitas, Ensenada 22860, Mexico; clarabarago@gmail.com (C.B.); ksidon@cicese.edu.mx (K.S.-C.); 4 Departamento de Innovación Biomédica, Centro de Investigación Científica y de Educación Superior de Ensenada, Carretera Ensenada-Tijuana No. 3918, Zona Playitas, Ensenada 22860, Mexico; 5 Instituto Gulbenkian de Ciência, Rua da Quinta Grande, 6, 2780-156 Oeiras, Portugal; rleite@igc.gulbenkian.pt

**Keywords:** type I PKS, bioactive compounds, biosynthesis of secondary metabolites, metagenomics, antibiotics production, marine sediments

## Abstract

The excessive use of antibiotics has triggered the appearance of new resistant strains, which is why great interest has been taken in the search for new bioactive compounds capable of overcoming this emergency in recent years. Massive sequencing tools have enabled the detection of new microorganisms that cannot be cultured in a laboratory, thus opening the door to the search for new biosynthetic genes. The great variety in oceanic environments in terms of pressure, salinity, temperature, and nutrients enables marine microorganisms to develop unique biochemical and physiological properties for their survival, enhancing the production of secondary metabolites that can vary from those produced by terrestrial microorganisms. We performed a search for type I PKS genes in metagenomes obtained from the marine sediments of the deep waters of the Gulf of Mexico using Hidden Markov Models. More than 2000 candidate genes were detected in the metagenomes that code for type I PKS domains, while biosynthetic pathways that may code for other secondary metabolites were also detected. Our research demonstrates the great potential use of the marine sediments of the Gulf of Mexico for identifying genes that code for new secondary metabolites.

## 1. Introduction

New antibiotics are urgently required to combat the emerging multi-drug resistant pathogens and new infectious agents [1]. In their natural environments, microorganisms produce a wide range of secondary metabolites with a variety of chemical structures [2]. As most of these microorganisms have never been cultured, identified, or classified, their great chemical richness remains unexplored [3]. However, this is changing as a result of the development of genomic techniques that do not depend on conventional cultivation, enabling the rapid progress of phylogenetic studies based on rDNA [4,5,6].

Secondary metabolites are produced by biosynthetic gene clusters, which are organized groups of two or more genes that encode a biosynthetic pathway to produce specialized metabolites [7]. Nonribosomal peptide synthetases (NRPSs) and polyketide synthases (PKSs) are two of the largest classes of biosynthetic gene clusters, encompassing most of the known antibiotics and antifungals [8]. With three types, I, II, and III, PKSs contain ketosynthase (KS) domains and a variety of other accessory domains, such as β-ketoacyl reductase (KR), dehydratase (DH), enoylreductase (ER), and methyltransferase (MT) [9,10]. Type I PKSs (PKS I) are multifunctional enzymes structurally organized into modules. An individual PKS enzyme can harbor one or multiple functional modules, each of which consist of several distinct active sites (domains) for each enzymatic step [10]. Type I PKSs can be further classified into modular or iterative classes, with the latter using the same domain many times, iteratively, to synthesize the polyketide. Modular PKSs are large multidomain enzymes in which each domain is used only once in the synthesis process [11,12].

Covering approximately 70% of the surface of the earth, the oceans are an invaluable source of new natural products [13]. With marine chemodiversity, one of the targets for the search for natural products as a source of new therapeutic drugs, multiple studies have been undertaken in this field to meet the growing demand for more effective antibiotics for combatting multiple diseases [14]. Analyzing the biodiversity of PKS I is an important tool for identifying new bioactive molecules capable of meeting the growing demand for more effective antibiotics. The study of these domains in marine sediments enables new PKS gene clusters to be identified, while ascertaining their abundance can facilitate the unlocking of their biosynthetic potential. The present study analyzes the main and accessory domains of PKS I in order to evaluate the potential of microbiomes from marine sediments of the Gulf of Mexico (GoM) to produce secondary metabolites.

## 2. Results

### 2.1. Metagenomic Reads Assembly and Coverage Analysis

Among the main problems encountered by the present study during the assembly of environmental samples were the low coverage and the formation of chimeras [15,16,17]. Assembling environmental samples is a complex task, despite the existence of multiple algorithms that can be used to minimize these problems. The search for biosynthetic genes can be an even more difficult process, as these genes generally contain repetitive domains that tend to be cut into multiple contigs [18]. We used the SPAdes genome assembler version 3.14.1 (metaSPAdes mode) [19,20] to assemble five metagenomes from marine sediments taken from the GoM, obtaining an average of 95,040 coding sequences from assemblies varying in size from 13.8 Mbp to 99.8 Mbp, and a relatively low N50. However, in all cases, N50 > 500 bp (~3.3× read length) (Table 1).

While the initial size of the assemblies was greater than 500 Mbp, after filtering with a contig cutoff >500 bp, the size decreased significantly, probably due to their high levels of fragmentation and low contiguity in them. The longest contigs corresponded to sample C13 and those with a size > 40 Kbp. However, these curated metagenomes still harbored more than 475,202 coding sequences with potential as novel bioactive compound pathways or functional elements. Raw data and a full quality assessment corresponding to the metagenome assemblies are shown in Supplementary Material S1.

Due to the drastic decrease in the contigs’ size, the coverage and diversity was evaluated based on the short reads, using the Nonpareil software [21,22], with the objective of assessing the fraction of the biomes represented in our data set. While the average coverage obtained from the short reads (which could represent the null model of diversity) was approximately 0.2 for all the metagenomes analyzed, the fraction of the diversity and richness captured in the assemblies is in line with the diversity indices estimated from the short reads (Table 2). This finding suggests that, although fragmented, the curated assemblies still capture a genomic space that effectively replicates the diversity contained in the short reads. The coverage, raw taxonomy profiles, and full diversity analysis are presented in Supplementary Material S2.

### 2.2. Screening PKS I Genes and Phylogenetic Analysis

**Figure 1:**
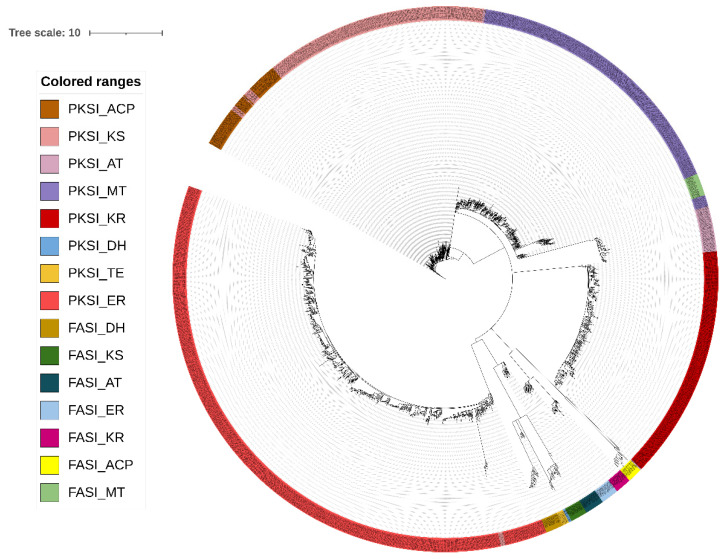
Maximum likelihood tree for the PKS type I domains. PKS I: type I PKS; FASI: fatty acid synthases; AT: acyltransferase; ACP: acyl carrier proteins; ER: enoyl reductase: KR: ketoreductase; KS: ketosynthase; MT: methyltransferase; DH: dehydratase; TE: thioesterase. The raw Phylip format tree file is presented in Supplementary Material S6.

The PKS I domains were identified by HMMer search using a predefined set of models (Supplementary Material S3). The search with Hidden Markov Models allowed to select 2066 candidate sequences coding for possible domains of PKS I in the sequences predicted by Prodigal (Supplementary Material S4). Of these candidate sequences, the most represented domain was ER, with 833 sequences, followed by the MT, KS, and KR domains, while the lowest values were obtained for the DH and TE domains. Due to the high similarity between PKS I sequences and fatty acid synthases (FAS I), we decided to complement the Hidden Markov Models with a phylogenetic study to confirm the right selection of the PKS I domains (Figure 1), using the sequences of the FAS I domain as the external group. FAS I sequences are available in Supplementary Material S5. Clustering in different clades for the domains that encode for PKS I and FAS I reaffirms the reliability of the method applied for selecting sequences that encode for biosynthetic genes.

**Figure 2:**
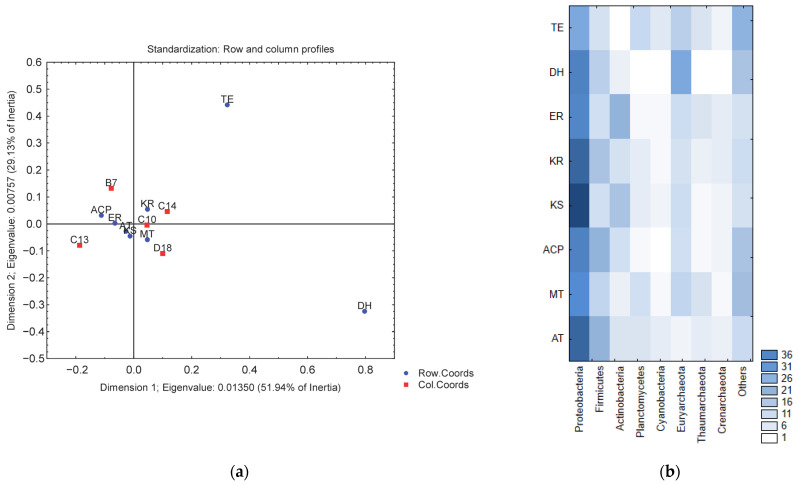
(**a**) Correspondence analysis between PKS I domains and metagenomes (red squares: metagenomes; blue dots: PKS I domains). (**b**) Representation of PKS I domains by relevant taxonomic groups.

**Figure 3:**
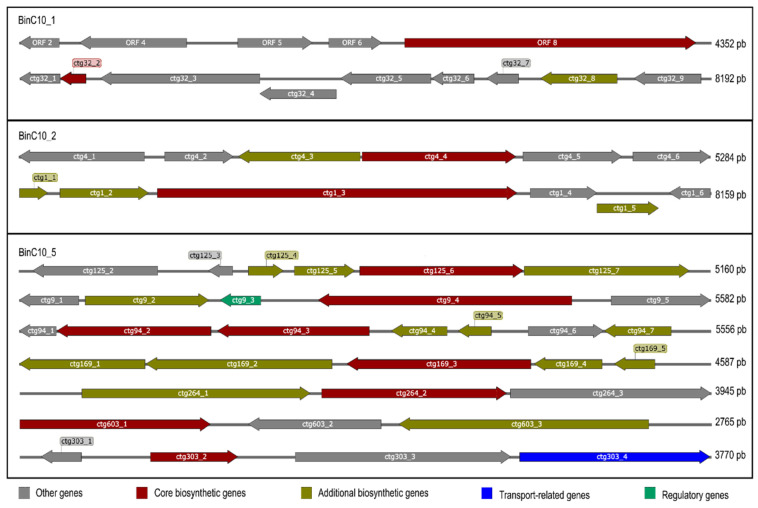
Biosynthetic clusters detected by antiSMASH bacterial version 6.0 in the BinC10_1, C10_2, and C10_5. The arrows presented within each open reading frame indicate the direction of translation for each gene. The type of product detected by AntiSMASH is presented above each core biosynthetic gene. Contig number: ctg; RiPP: ribosomally synthesized and post-translationally modified peptides; RRE: RiPP recognition element; NRPS: nonribosomal peptide synthetases; T3PKS: type III PKS.

The normalization of the data and correspondence analysis indicate that the KS, KR, MT, ER, ACP, and AT domains are related (clustering close each other), while the TE and DH domains presented a minor relative abundance in our data (the TE domain was not found in the B7 and C13 metagenomes) (Figure 2). The distribution of these domains by taxonomic group (Figure 3) showed that the greatest diversity of PKS I domains belongs to the phyla *Proteobacteria* and *Firmicutes*, while, in the kingdom of *Archaea* the phylum *Euryarchaeota* is the most common carrier of these domains. The FOCUS taxonomic profiling results for contigs containing PKS I domains are shown in Supplementary Material S7.

### 2.3. Marine Sediments of the GoM as a Source of Bioactive Compounds

The synthesis of secondary metabolites by bacteria helps to defend against predators and enables cell communication, among other functions, making secondary metabolites an excellent source of bioactive compounds for use in human therapies [23]. Deep-water marine sediments are a source of bioactive compounds that remain unexplored, due to the technical challenges of collecting the samples and the large area they occupy. The domains found in the five metagenomes obtained from sediments taken from the GoM were compared against the KEGG database to identify orthologues that may be involved in the synthesis of secondary metabolites. The results enabled the identification of 203 KO ortholog sequences (Table 3) involved in at least 14 metabolic pathways of bioactive synthesis (Supplementary Material S8).

The most represented metabolic pathway was the biosynthesis of prodigiosin, an antimicrobial agent with little toxicity. Another pathway detected in the metagenomes obtained was the production of monoterpenoid, which consists of a ten-carbon backbone (two isoprene units) structure and can be divided into three subgroups: acyclic, monocyclic, and bicyclic [24]. Some monoterpenes have been described as presenting antimicrobial properties and painkilling effects [25]. Another compound detected was acarbose, an alpha-glucosidase inhibitor, which is described as a group of poorly absorbed antidiabetic agents [26]. Finally, domains related to the production of antibiotics, such as ansamycins, enediyne, vancomycin, streptomycin, and validamycin, were also present.

### 2.4. Exploring Biosynthetic Genes from Genome Bins

After binning was performed, the metagenome-assembled genomes (MAGs) were analyzed using the antiSMASH software, bacterial version 6.0, to find biosynthetic clusters. While most of the clusters obtained were incomplete, due to the short length of the contigs obtained, we were able to detect domains involved in the synthesis of the bioactive compounds from our samples.

The assembly of the C10 metagenome presented the highest quality (Table 1), in that it enabled the detection of several possible biosynthetic clusters. We were able to deconvolve three MAGs from the C10 metagenome: BinC10_1 (2 Mb in size and 73.74% completeness); BinC10_2 (700 Kb in size and 25% completeness); and BinC10_5 (2.9 Mb in size and 78.67% completeness) (Supplementary Material S9). Sequences related to ribosomally synthesized and post-translationally modified peptides (RiPPs) (Figure 3) were found in the BinC10_1, which was taxonomically consistent with the *Desulfobacteraceae* family (genomic Mash distance 0.07). The BinC10_2 contained domains that may code for NRPS and terpene, while BinC10_5 presented both the highest abundance of biosynthetic genes, which code for the RiPP recognition element (RRE), and the synthesis of terpene, ladderane, and type III PKS.

## 3. Discussion

Type I PKS produces a large family of medicinally important natural products. As PKS I multidomain proteins can be long and present a high degree of complexity, in most metagenomic sequences, the fragments of these proteins contain a simple domain [27]. The low number of multidomains found in our data could be due to the low level of metagenome coverage presented by the samples. However, by means of the use of Hidden Markov Models and phylogenetic relationships to facilitate the search for PKS, the present study was able to show the potential of these microbiomes to produce bioactive compounds from marine sediments. Each of the domains identified in the metagenomes found by the present study are likely to represent an entire PKS protein. Foerstner et al. (2008) built Hidden Markov Models to find PKS I domains, identifying PKS I domains from different metagenomes and annotating multiple proteins of unknown function in the UniRef database [27].

An average coverage (close to 0.2) consistent with complex and highly diverse communities (such as those found in marine sediments) was observed in our data set. The statistics obtained by the present study coincide with metagenomic observations obtained by other studies in complex environmental samples, such as soil, tropical forest, or seawater. Said studies obtained coverage levels that were always < 40%, which coincides with the greater level of diversity found in their samples than in other biomes, such as animal host microbiomes or enriched communities whose coverage has been found to be >60% by similar sequencing efforts [22,28]. Therefore, although coverage in metagenomics is still an important feature to consider, this metric depends more on the nature of the biome sampled than the data size [28]. We argue that the complexity of the communities represented in metagenomes B7 to D18 may influence the coverage values obtained by the present study. Moreover, we evaluate the diversity and richness indices as estimators of the number of species present in the samples, their distribution, and its representativeness. In all cases, the diversity and richness indices captured for the assemblies were not significantly different from those estimated from the short reads (Table 2). This indicates that the information contained in the assemblies captures the information from the null model at the taxonomic level, although the assembly is not expected to always express the entire diversity space of the entire sample (see gray columns in Table 2, wherein the closer the ratio is to 1, the more representative the metagenomic assembly). Finally, the Nonpareil diversity index concurs with those obtained by other studies for marine and other sandy soil communities, with expected values ranging from 21 to 25 (http://enve-omics.ce.gatech.edu/nonpareil/faq (accessed on 25 May 2022): B7 = 23.17; C10 = 23.05; C13 = 23.03; C14 = 22.92; and D18 = 22.96). This finding also supports the conclusion that the genomic space captured in our data is representative of the type of biomes analyzed.

Degenerate primers are usually used to identify KS and ACP domains in biodiversity studies or for the identification of new biosynthetic clusters [29], which limits the information available on the rest of the domains, especially the DH and TE domains. The correspondence analysis conducted by the present study indicates a limited presence of said domains (DH and TE) in the sediments and a lower ratio of the remaining domains of interest (KS, ACP, AT, ER, KR, and MT). Foerstner et al. (2008) identified PKS I domains in six metagenomes and the UniRef database, finding that, of the total domains identified (22,106), only 6.7% and 2.4% corresponded to DH and TE, respectively. This finding reflects either the low abundance of these domains in bacterial biosynthetic clusters or the scarcity of information about said domains, which presents a challenge to being able to identify them more reliably [27].

Our results are consistent with the biodiversity studies carried out in the GoM, which have found that its sediments largely contain the phyla *Proteobacteria*, *Firmicutes*, *Actinobacteria*, *Plantomycetes*, and *Cyanobacteria* [30,31], which have a proven potential as producers of bioactive compounds [32,33,34]. However, a large number of natural products have been isolated in *Actinobacteria* [23,35], and more than half of the KS genes in the Uniprot database belong to *Actinobacteria* [36,37]. The genus *Streptomyces* continues to be the predominant source of new chemistries, with 167 new metabolites reported during 2018, representing >69% of the marine-sourced bacterial natural products [38], although in recent years marine bioactives have been reported in other species, such as *Roseovarius tolerans* [39], *Rhodovulum sulfidophilum* [40], and *Aequorivita* sp. [41]. It cannot be ruled out that the reported predominance of *Streptomyces* is due to the fact that they are culturable microorganisms and they are highly represented in annotation databases. The phylum found by the present study to present the highest number of PKS I domains is *Proteobacteria*, which is the most abundant phylum found in marine environments (with an abundance between 50% and 80%); however, very few bioactive compounds have been described in these microorganisms [23].

The taxonomic profiling carried out by our study found that *Actinomycetales*, *Clostridiales*, *Planctomycetales*, *Rhizobiales*, and *Spirochaetales* are the orders that present more than two PKS domains in their sequences. While *Clostridiales* presents limited natural products, the genomic analysis conducted on these strict anaerobes shows the presence of natural product biosynthetic gene clusters that can code for entirely new products [42]. Graça et al. (2016) evaluated the genome of 40 taxa of *Planctomycetales* isolated from macroalgae obtained off the Portuguese coast, finding that 95% contained one or both of the bioactive genes PKS and NRPS; in addition, they also found antifungal and antibacterial activity in the bioactivity tests conducted on the samples [43]. The order *Rhizobiales* includes species of the genera *Agrobacterium*, *Blastobacter*, *Mesorhizobium*, and *Ochrobactrum*, which have been associated with bioactive compounds of marine origin, in contrast with *Spirochaetales,* for which no marine bioactive metabolites have been reported in the Comprehensive Marine Natural Products Database [44].

Prodigiosin is one of the secondary metabolites that are encoded in the metagenomes analyzed in this study. This red tripyrrole pigment, belonging to the prodiginines family, is produced by several bacterial genera, such as *Serratia*, *Hahella*, *Streptomyces*, *Zooshikella*, *Vibrio*, and *Pseudomonas* [45], and is known to have immunosuppressive, antifungal, antiviral, antimicrobial, anti-malarial, and anti-proliferative properties [46,47]. Genes encoding monoterpenoids were also detected in our data. The relatively low molecular weights of monoterpenoids and their intrinsic lipophilicity make these molecules suitable for administration via skin permeation and potential candidates for use in topical treatments, especially those used to relieve chronic pain [25]. Moreover, they have also been associated with various antimicrobial, hypotensive, anti-inflammatory, and antipruritic functions, among others [48]. Of the antidiabetic drugs, acarbose is associated with lesser gastrointestinal side effects than other alpha-glucosidase inhibitors and has been used as a single drug or in combination with other antidiabetic medications to control blood glucose levels in type 2 diabetic patients [26].

Among the antibiotic biosynthesis pathways found in the metagenomes analyzed are ansamycins, enediyne, vancomycin, streptomycin, and validamycin. Ansamycins are a group of antibiotics produced by several *Actinomycetes* strains and are molecules that have been proven to have very potent anticancer, antibacterial, and antiviral effects [49]. Enediyne natural products are among the most cytotoxic natural products ever discovered and are a promising source of next-generation antibody–drug conjugate payloads [50]. Vancomycin is enlisted as a drug of last resort for the treatment of resistant Gram-positive bacterial infections and is the first-choice antibiotic for the treatment of methicillin-resistant *S. aureus* infection [49].

The first of the class of aminoglycoside antibiotics to be discovered was the first antibiotic remedy for tuberculosis [49] and was derived from the Gram-positive bacteria of the genus *Streptomyces* [51]. Streptomycin is a broad-spectrum drug effective against both Gram-negative aerobic bacteria and staphylococci. Another aminoglycoside compound is validamycin [52], which has been used to control the rice sheath blight caused by *Rhizoctonia solani* for over 50 years in China, with no validamycin-resistant isolates reported in the field [52,53].

The search for biosynthetic clusters in the metagenome bins found the presence of RiPPs. Research conducted in the 20th century identified many classes of natural products, with four groups being particularly prevalent (terpenoids, alkaloids, polyketides, and non-ribosomal peptides), while, more recently, RiPPs have also been described [54]. RiPP biosynthesis starts with the ribosomal synthesis of a linear precursor peptide, and many RiPP biosynthetic proteins recognize and bind their cognate precursor peptide via a domain known as the RiPP recognition element (RRE) [55,56]. The various RiPPs that present antibiotic activity are widely addressed by Hudson and Mitchell (2018) [57]. Sequences encoding for RiPPs and RRE were found in BinC10_1, which belongs to the *Desulfobateraceae* family and represents a new species (MASH distance 0.07) with an unexplored genomic context.

Several biosynthetic clusters have also been detected in BinC10_5: RRE, terpene, ladderane, and type III PKS. While the terpene biosynthesis pathway is well known to be present in many plant and fungi genomes, it was recently proposed that it is also widely distributed in bacterial genomes [58]. Largely found as constituents of essential oils, terpenes are mostly hydrocarbons [59], and have been associated with medicinal and therapeutic properties such as those harnessed for anti-inflammatory therapies and the treatment of malaria, bacterial infections, and cardiovascular diseases [60]. Ladderane is exclusively present in the membranes of anaerobic ammonia-oxidizing (anammox) bacteria [61], which are able to oxidize ammonia via nitrite reduction into nitrogen gas. This process takes place in a separate intracytoplasmic organelle called the anammoxosome, which presents a high concentration of ladderane lipids that makes the membrane less permeable and, thus, provide a tight barrier against diffusion. This is assumed to be an important feature for retaining toxic intermediates, such as hydrazine (N_2_H_4_) and hydroxylamine (NH_2_OH), within the anammoxosome [62]. The identification of BinC10_5 using the Mash software has shown that this genome probably pertains to the *Candidatus* Scalindua genus (Mash distance 0.2), a genus described from natural habitats, especially from marine sediments and oxygen minimum zones [61,63,64]. *Candidatus* is a taxonomic status for uncultured prokaryotic cells [63], therefore it is likely that many of the genes detected in the present study code for entirely new antimicrobials.

Previous studies show that geographic location, latitude, and pH are determining factors in the diversity of biosynthetic genes and environmental microbiomes [64,65,66]. The GoM is one of the marine ecosystems most affected by the uncontrolled extraction of hydrocarbons and countless oil spills [67], which is why its bacterial biodiversity is distinctive and largely influenced by environmental factors specific to the region, especially to the presence of hydrocarbons [68]. The natural conditions of the ocean (low temperatures, high salinity, and high pressure), together with the hydrocarbon contamination, favor the natural selection of polyextremophilic microorganisms, which have gained the attention of biotechnologists due to their metabolic diversity and their ability to produce secondary metabolites. The marine sediments of the GoM are undoubtedly an important source of secondary metabolites to consider if we want to obtain new antimicrobials to combat the emerging resistant strains.

## 4. Materials and Methods

### 4.1. Sampling Sites and DNA Sequencing

The sediments were collected onboard the R/V Justo Sierra (UNAM) during the MMF-01 oceanographic campaign from 25 February to 18 March 2016. Eighteen sampling sites were selected from the Perdido and Coatzacoalcos regions and equally distributed between the two. Sampling was undertaken at a seafloor depth ranging from 550 to 3400 m using a box core, with subsampling then directly obtained from the box core using sterile syringes inserted at a depth of up to 10 cm. Each subsample was frozen and stored immediately in liquid nitrogen onboard and kept at −80 °C when they arrived to the laboratory, until nucleic acid extraction could be performed. For this study the results of the 16S-rDNA sequencing (see [69]) correspond to one sample from the Perdido region (B7, 1200 m) and the rest from the Coatzacoalcos region (C10, 550 m; C13, 2500 m; C14, 3200 m; and D18, 1500 m).

DNA was extracted from three independent syringes from each sampling site using the DNeasy PowerSoil Kit (Qiagen^®^, Hilden, Germany) following the protocol provided by manufacturer, with some modifications. During the lysis step, 275 µL of phenol-chloroform-isoamyl alcohol (25:24:1) solution was added, followed by 5 min incubation at room temperature to increase cell lysis. The elution step was performed twice using 50 µL of elution buffer with the column incubated for 10 min at room temperature. All centrifuge steps were performed at 14,000× *g*. Eluted DNA was quantified via UV absorption analysis (NanoDrop 2000 Spectrometer, Thermo Fisher Scientific, Waltham, US) and the quality of the extracted DNA was verified on an agarose gel electrophoresis. DNA was stored at −20 °C until further analysis.

Paired-end metagenome sequencing was performed in a NovaSeq instrument (2 × 150 bp) at MR DNA (Molecular Research LP, Shallowater, TX, USA).

### 4.2. Quality Control and Assembly

The quality of the short reads (151 pb) was assessed using the FastQC software version v0.11.5 and low-quality bases (Q < 30) and adapters were removed using AfterQC version 0.9.6, which was configured with the paired-end mode and default options [70]. The reads were assembled via SPAdes genome assembler v3.14.1 (metaSPAdes mode)—only-assembler option [20]. After contigs assembly, only contigs with a length of more than 500 bp were retained (3.3× read length). The quality of the assembly was evaluated with Quast Version: 5.0.2 option Meta [71]. The coding sequences were obtained with PRODIGAL v2.6.3 [72]. The taxonomic profiling of the contigs was performed on FOCUS using the default reference database [73].

### 4.3. Taxonomy Profiles, Coverage, and Diversity Estimations

The five metagenomes reported in the present study were profiled from both the short reads (null model for diversity) and the curated assemblies using the FOCUS software [73] with a enriched custom database comprising 14,551 genomes retrieved from the Assembly Database on the National Center for Biotechnology Information (NCBI). The genomes had to pertain to the type material in the NCBI database, in order to satisfy the current nomenclature standards [74]. This new representative database is available at https://github.com/ayixon/RaPDTool (accessed on 25 May 2022). The relative abundance profiles using FOCUS were directly loaded in the web biodiversity calculator (https://alyoung.com/labs/biodiversity_calculator.html, accessed on 25 May 2022), to obtain the Shannon index, the Equitability index, and the Margalef Richness index. The metagenome coverage was estimated using the Nonpareil software version 3.401 [21,22], applying the kmer algorithm on one of read files (R1 sister). The coverage curves were generated with the R functions Nonpareil.curve and Nonpareil.set.

### 4.4. Hidden Markov Model Search and Phylogenetic Tree Reconstruction

The Hidden Markov Models for each of the PKS I domains (acyltransferase (AT), acyl carrier proteins (ACP), enoyl reductase (ER), ketoreductase (KR), ketosynthase (KS), methyltransferase (MT), dehydratase (DH), and thioesterase (TE)) were downloaded from the Pfam database, version 33.1 [75]. The models were used to find protein sequences containing these domains with HMMER 3.3.2 (hmmsearch with *--cut_tc* option “trusted cutoff (TC)”) [76]. Sequences with a score greater than 40 and an E-value below 10^−4^ were selected and aligned using MAFFT v7.453 [77]. The alignments were manually curated, and the phylogenetic tree was built with IQ-TREE, multicore version 1.6.12, with the best-fit model WAG + F + R5, which was chosen according to the Bayesian Information Criterion [78,79]. The tree visualization was conducted using the iTOL tool [80].

### 4.5. Bioactive Potential in Marine Sediments and Environmental Draft Genome Reconstruction

The Kofam_scan tool, version to 1.3.0 (https://github.com/takaram/kofam_scan, accessed on 25 May 2022) [81], was applied on the five marine metagenomes to search for orthologues that may be involved in the synthesis of secondary metabolites. Putative biosynthesis pathways were explored using Kegg Mapper Server [82]. Draft environmental genomes (bins) were also constructed from the five metagenomes independently using Metabat2 [83] and then surveyed using AntiSMASH, bacterial version 6.0 [84], in order to detect the gene clusters that code for bioactive compounds. The bins were cured with RefineM [85], while their quality was analyzed using CheckM workflow: checkm lineage_wf [86]. The bins were identified via genomic comparison against the Genome Taxonomy Database, release [87].

## Figures and Tables

**Table 1:** Results for the assembly of metagenomes from marine sediments of the GoM.

Item	B7 (1200 m)	C10 (550 m)	C13 (2500 m)	C14 (3500 m)	D18 (1500 m)
Metagenome Assembly Data					
Sequencing technology	Illumina 2 × 150 bp				
Assembly method	SPAdes assembler (metaSPAdes mode)				
No. of contigs	22,110	122,996	31,210	57,793	97,171
N50	591	775	633	712	684
N75	535	605	551	575	566
L50	9237	40,525	12,420	20,421	33,483
L75	15,410	77,347	21,277	37,504	62,922
Metagenome Features					
Size (>0 bp)	533,840,363	551,431,092	431,618,439	521,418,371	756,722,373
Size (≥500 bp)	13,832,311	99,792,104	20,782,953	43,271,384	72,530,889
GC content (%)	55.6	57.88	56.69	57.91	50.97
No. of putative total coding sequences	28,617	188,692	42,167	81,739	133,987
Longest sequences (bp)	6143	21,536	42,054	7539	43,361

The analysis reported in this manuscript is based on assemblies with contigs size ≥ 500 bp.

**Table 2:** Diversity indicators estimated from the metagenomic reads (null diversity model) and from the metagenomic assemblies.

	Shannon Index			Equitability Index			Margalef Richness Index		
	Short Reads	Assembly	Ds/Da	Short Reads	Assembly	Es/Ea	Short Reads	Assembly	Rs/Ra
B7	3.57	3.39	0.95	0.97	0.95	0.97	1.52	1.41	0.93
C10	3.27	3.36	1.03	0.96	0.95	0.98	1.17	1.38	1.18
C13	3.44	3.40	0.99	0.95	0.90	0.95	1.44	1.44	1.00
C14	3.49	3.39	0.97	0.96	0.95	0.99	1.48	1.40	0.95
D18	3.28	3.07	0.94	0.95	0.93	0.98	1.22	1.07	0.88

Ds: Short reads diversity; Da: assembly diversity; Es: short reads equitability; Ea: assembly equitability; Rs: short reads richness; Ra: assembly richness.

**Table 3:** Orthologues with possible biosynthetic function found when evaluating the PKS I domains present in the metagenomes of marine sediments of the GoM.

Biosynthesis of Secondary Metabolites	Number of Sequences	Metagenome Sample	PKS Domain	Orthology	Definition
Monoterpenoid biosynthesis	1	C14	KR	K15095	(+)-neomenthol dehydrogenase
Type I polyketide structures	2	D18	KR	K15643	myxalamid-type polyketide synthase MxaB
			AT	K16410	stigmatellin polyketide synthase StiF
	2	C10	ACP	K16025	methoxymalonate biosynthesis acyl carrier protein
			KR	K16417	myxalamid-type polyketide synthase MxaC
	1	C14	KR	K20788	myxalamid-type polyketide synthase MxaE
Biosynthesis of ansamycins	1	C10	ACP	K16025	methoxymalonate biosynthesis acyl carrier protein
Biosynthesis of enediyne antibiotics	8	C10	AT KS KR	K15314	enediyne polyketide synthase
	6	D18	DH KR KS		
	1	C13	KS		
	2	C14	KS		
	1	C10	ATC	K15320	6-methylsalicylic acid synthase
	1	C10	MT	K21172	enediyne biosynthesis protein CalE5
	2	C13	MT		
	3	C14	MT		
Biosynthesis of type II polyketide backbone	1	C10	ACP	K05553	minimal PKS acyl carrier protein
Tetracycline biosynthesis	1	C10	ACP	K05553	minimal PKS acyl carrier protein
Polyketide sugar unit biosynthesis	1	C13	ER	K01710	dTDP-glucose 4,6-dehydratase
Nonribosomal peptide structures	1	B7	ACP	K15654	surfactin family lipopeptide synthetase A
	2	C10	ACP		
	2	C13	ACP		
	2	B7	ACP KS	K15661	iturin family lipopeptide synthetase A
	1	C10	KS		
	1	D18	KS		
	1	C13	ACP	K15665	plipastatin/fengycin lipopeptide synthetase B
	1	C14	ACP	K15667	plipastatin/fengycin lipopeptide synthetase D
	1	D18	ACP		
Biosynthesis of siderophore group nonribosomal peptides	2	B7	ACP TE	K02364	L-serine-[L-seryl-carrier protein] ligase
	6	C10	ACP TE		
	2	C13	ACP		
	5	C14	ACP TE		
	4	D18	ACP TE		
	5	C10	ACP	K04780	glyine-[glycyl-carrier protein] ligase
	2	C13	ACP		
	1	C14	ACP		
	1	D18	ACP		
Biosynthesis of vancomycin group antibiotics	1	C13	ER	K01710	dTDP-glucose 4,6-dehydratase
Streptomycin biosynthesis	1	C13	ER	K01710	dTDP-glucose 4,6-dehydratase
Acarbose and validamycin biosynthesis	1	C13	ER	K01710	dTDP-glucose 4,6-dehydratase
Prodigiosin biosynthesis	7	B7	KR	K00059	3-oxoacyl-[acyl-carrier protein] reductase
	23	C10	KR		
	6	C13	KR		
	21	C14	KR		
	17	D18	KR		
	4	B7	AT	K00645	[acyl-carrier-protein] S-malonyltransferase
	16	C10	AT		
	5	C13	AT		
	5	C14	AT		
	16	D18	AT		
	1	C14	KS	K21783	beta-ketoacyl ACP synthase
	1	C14	ACP	K21784	4-hydroxy-2,2’-bipyrrole-5-methanol synthase
	1	C13	ACP	K21790	acyl carrier protein
	1	C14	ACP		
Biosynthesis of various secondary metabolites	1	D18	ACP	K02078	acyl carrier protein
	1	C10	ACP		

## Data Availability

The data presented in this study are available in both the article and in the Supplementary Materials.

## Supplementary Materials

Supplementary files can be found through the link: https://doi.org/10.6084/m9.figshare.19769023 (accessed on 16 May 2022). File S1: Assembly of the different marine sediment samples obtained from the GoM; File S2: Taxonomy profiles, coverage, and diversity estimations; File S3: Hidden Markov Models (HMM) used to identify PKS I domains in metagenomes; File S4: Domain sequences identified by HMM in metagenomes; File S5: Sequences of fatty acid synthase (FAS) domains used as control or outer group in phylogeny; File S6: Phylogenetic tree file: File S7: Taxonomic profiling results for contigs containing PKS I domains, File S8: Orthologous search results with Kofamscan, File S9: Metagenome-assembled genomes BinC10_1, BinC10_2, BinC10_5.
